# Selecting Administration Routes for Therapeutic Extracellular Vesicles to Achieve Optimal Drug Delivery

**DOI:** 10.3390/molecules31203725

**Published:** 2026-10-17

**Authors:** Minah Lee, Yeseul Choi, Sung-Jin Yoon, Minseong Kim

**Affiliations:** 1 Department of Biopharmaceutical Engineering, College of Life Science and Nanotechnology, Hannam University, Daejeon 34054, Republic of Korea; lma040909@naver.com (M.L.); cvb4868@naver.com (Y.C.); 2 Department of Functional Genomics, KRIBB School of Bioscience, University of Science and Technology (UST), Daejeon 34113, Republic of Korea; 3 Environmental Disease Research Center, Korea Research Institute of Bioscience and Biotechnology (KRIBB), Daejeon 34141, Republic of Korea

**Keywords:** extracellular vesicles, administration routes, drug delivery

## Abstract

Extracellular vesicles (EVs) have emerged as promising therapeutic carriers owing to their biocompatibility, low immunogenicity, and ability to transport diverse biological cargo. However, successful EV-based therapy depends not only on carrier engineering but also on selecting an administration route appropriate for the target disease. This review examines major administration routes for therapeutic EVs, with lipid nanoparticles (LNPs) as a comparative platform, and discusses how anatomical barriers, biodistribution, clearance, inflammatory microenvironments, and target-cell localization influence delivery efficiency. Systemic administration provides broad accessibility but often results in substantial off-target distribution, whereas localized or barrier-bypassing routes can enhance exposure at specific pathological sites. Importantly, anatomical proximity does not necessarily guarantee efficient delivery, highlighting the need for quantitative assessment of target-tissue bioavailability. We propose a shift from a carrier-centered toward a disease- and route-centered paradigm integrating disease pathology, anatomical accessibility, biodistribution, and carrier engineering.

## 1. Introduction

The field of pharmaceutical biotechnology has witnessed a profound paradigm shift in the understanding and application of extracellular vesicles (EVs). Long dismissed as mere physiological debris or cellular waste-disposal mechanisms—metaphorically referred to as “rubbish bags” for the cell—EVs are now recognized as essential mediators of sophisticated intercellular communication [1,2,3]. These lipid bilayer-delimited nanoparticles are secreted by virtually all prokaryotic and eukaryotic cell types studied to date and are ubiquitous in human biofluids, including blood, breast milk, saliva, semen, and urine [1,4,5].

The structural and functional complexity of EVs arises from their diverse cargo, which includes proteins, lipids, and various species of nucleic acids such as mRNA, miRNA, and lncRNA [6,7,8]. This composition reflects the physiological or pathological state of the parent cell, positioning EVs not only as potent therapeutic agents but also as high-resolution diagnostic biomarkers for liquid biopsies [5,7,9]. As the biopharmaceutical industry seeks cell-free alternatives to traditional regenerative medicine, EVs offer a compelling profile characterized by intrinsic stability, low immunogenicity, and superior biocompatibility compared to synthetic lipid nanoparticles or cell-based therapies [3,6,10].

The maturation of this field is encapsulated in the evolution of standardized guidelines. The International Society for Extracellular Vesicles (ISEV) has refined the “Minimum Information for Studies of Extracellular Vesicles” (MISEV) guidelines, with the 2023 iteration (MISEV2023) emphasizing rigor, reproducibility, and transparency [9,11]. These guidelines move away from nomenclature based purely on biogenesis—such as “exosomes” originating from the endosomal system and “microvesicles” from plasma membrane budding—favoring more precise classification based on size (small EVs vs. medium/large EVs), density, or biochemical markers [6,12]. This standardization is a critical prerequisite for the clinical translation of EV-based drug delivery systems (DDS), as it addresses the inherent heterogeneity of these biological particles [13].

The biological landscape of EVs is categorized into three primary subtypes defined by their biogenic pathways and physical dimensions. Exosomes, typically ranging from 30 to 150 nm in diameter, are formed through the inward budding of the endosomal membrane to create intraluminal vesicles within multivesicular bodies (MVBs), which subsequently fuse with the plasma membrane for release. Microvesicles (MVs), also known as ectosomes, are larger particles (100 to 1000 nm) produced by direct outward blebbing and fission of the cell’s plasma membrane. Apoptotic bodies (ApoBDs) are the largest category (1000 to 5000 nm), generated during the process of programmed cell death [1,3,5,6].

In the context of therapeutic delivery, small EVs (sEVs) have garnered the most attention due to their ability to navigate complex physiological barriers and their favorable pharmacokinetic profiles [4,14]. The integration of these vesicles into clinical workflows necessitates an exhaustive understanding of how different administration routes influence their biodistribution, half-life, and ultimate therapeutic efficacy [6].

In this review, we will use the term “extracellular vesicles” (EVs) unless their specific biogenesis pathways have been established, except when referring to size-defined EV populations or retaining the terminology used in the original clinical studies.

Here, we will navigate various drug administration routes and their pros and cons. In addition, we will review and discuss which routes should be considered to elicit better pharmaceutical efficiency, focusing on EVs and LNPs. Furthermore, we will touch on the current challenges and hurdles of EV- or LNP-based drug delivery, and the efforts to mitigate those challenges.

## 2. Results

### 2.1. Various Routes for Drug Administration

#### 2.1.1. Intravenous Injection (IV)

**Figure 1:**
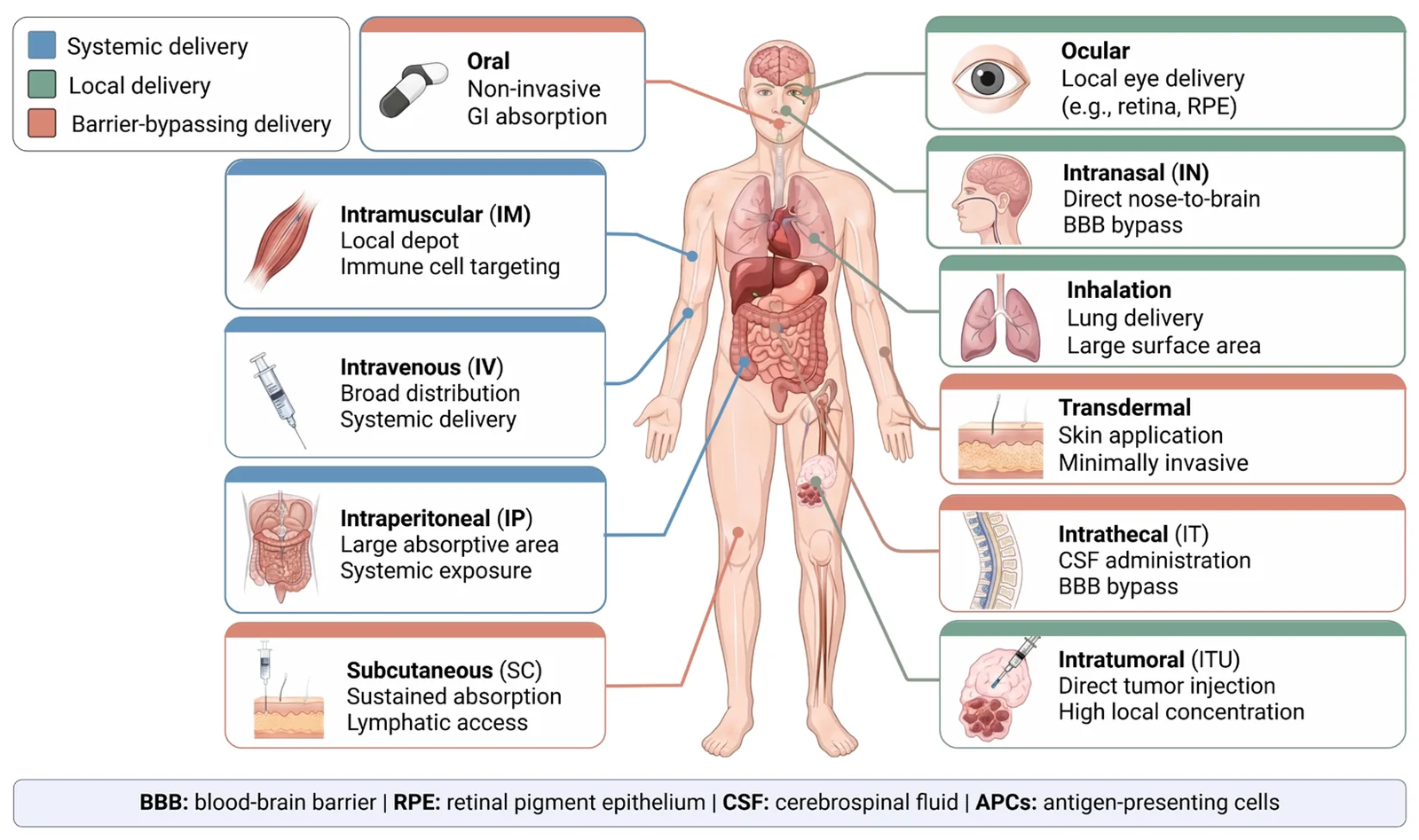
Administration routes for therapeutic EVs and LNPs. Schematic overview of major administration routes used for the delivery of therapeutic EVs and LNPs. These routes were categorized into systemic delivery (blue), local delivery (green), and barrier-bypassing delivery (red), according to their primary delivery characteristics. The optimal administration route depends on the target tissue, desired biodistribution, local bioavailability, and anatomical or physiological barriers. BBB, blood–brain barrier; RPE, retinal pigment epithelium; CSF, cerebrospinal fluid; APCs, antigen-presenting cells. Created in BioRender (https://www.biorender.com/).

Since direct intravascular injection allows for systemic circulation and subsequent multi-organ targeting, intravenous (IV) administration serves as the primary route for both LNPs and EVs. Consequently, IV injection is widely utilized as the standard reference in targeting studies for the CNS, liver, and tumors. Intravenous (IV) administration enables immediate delivery into the systemic circulation, allowing EVs to distribute to multiple organs, including the liver and tumors (Figure 1).

Intravenous injection is an LNP’s representative route for systemic targeting of the liver, spleen, and tumors. IV delivery serves as the core platform for mRNA-LNP therapeutics and vaccines. While LNPs offer high mRNA delivery efficiency and allow for straightforward surface modification with targeting ligands, they face limitations such as excessive accumulation in the liver and potential PEG-associated immune responses.

EVs are widely investigated via the IV route as a standard systemic therapy to target multiple organs, including the heart, brain, liver, and kidneys. EVs exhibit significant immune evasion and favorable safety upon repeated administration, alongside a natural biodistribution to the liver and kidneys [15]. However, their therapeutic efficacy can be hindered by rapid hepatic clearance and relatively weak targeting capability [16].

#### 2.1.2. Intraperitoneal Injection (IP)

Since direct intraperitoneal injection allows for localized exposure within the abdominal cavity and subsequent systemic absorption, intraperitoneal (IP) administration serves as a valuable route for both LNPs and EVs in specialized contexts. Consequently, IP injection is widely utilized as a standard reference in targeting studies for the peritoneum, visceral organs, and abdominal tumors. Intraperitoneal (IP) administration enables direct delivery into the peritoneal cavity, potentially increasing local exposure to the peritoneal wall and intra-abdominal tumors (Figure 1).

Lipid nanoparticles (LNPs) are employed via the IP route in selected preclinical models for cancer and gene therapies. Intraperitoneal injection is an LNP’s representative route for localized and regional targeting of the ovaries, pancreas, and peritoneal carcinomatosis. IP delivery serves as a core platform for mRNA-LNP therapeutics targeting intra-abdominal tumors [17,18,19,20]. While LNPs offer high mRNA delivery efficiency within the peritoneal cavity and allow for straightforward surface modification with targeting ligands, they face limitations such as rapid clearance from the abdominal space into systemic circulation and potential PEG-associated immune responses.

EVs are extensively investigated through IP delivery, particularly in preclinical models of peritoneal metastasis and intraperitoneal inflammation. EVs are widely investigated via the IP route to show multiple tissues targeting within the abdominal cavity, including the peritoneum, mesenteric lymph nodes, and visceral organs [21,22]. EVs exhibit favorable immune evasion and a high safety profile upon repeated administration, alongside a natural biodistribution to the liver and spleen after systemic absorption. However, their therapeutic efficacy can be hindered by rapid lymphatic drainage from the peritoneal cavity and relatively weak targeting capability.

#### 2.1.3. Subcutaneous Injection (SC)

The subcutaneous injection route facilitates lymphatic targeting, making it highly suitable for immunomodulation, and allows for sustained absorption to achieve prolonged therapeutic effects (Figure 1).

Due to their advantages in targeting immune cells and lymph nodes, lipid nanoparticles (LNPs) are frequently investigated in vaccine development and immunomodulatory therapies. LNPs offer high mRNA loading efficiency and robust manufacturing standardization.

EVs are utilized via this route for treating chronic inflammation and immune disorders, as well as for achieving systemic therapeutic effects. A key advantage of EVs is their high biocompatibility, which ensures safety upon repeated administration [23].

#### 2.1.4. Intramuscular Injection (IM)

Intramuscular administration serves as the standard for vaccine delivery, allowing for effective localized uptake and subsequent systemic dissemination from the muscle tissue. In addition, this administration route enables immediate deposit into the skeletal muscle, facilitating efficient cellular uptake and long-lasting antigen presentation or therapeutic release. Therefore, this route is highly established as the representative delivery pathway for mRNA vaccines, most notably demonstrated by COVID-19 mRNA-LNP formulations (Figure 1).

LNPs administered via the IM route initially had issues, including liver accumulation. Later, this was mitigated by the development of LNPs with an albumin-binding module on the surface, which leads LNPs to a lymphatic vessel. It is expected to offer a a better efficacy for the LNP-based mRNA vaccine [24].

EVs s are currently being investigated via IM delivery for their therapeutic potential in muscle regeneration, sarcopenia, and as novel vaccine platforms. Beyond their role as a delivery vehicle, EVs provide inherent therapeutic benefits for muscle repair with a lower risk of localized inflammation [25].

#### 2.1.5. Transdermal Delivery (TD)

Transdermal delivery can be classified into invasive approaches such as with microneedles, and non-invasive approaches, such as with patches and topical creams. Both approaches can be used for localized and systemic drug delivery. TD delivery has emerged as a patient-friendly, minimally invasive route of administration that bypasses hepatic first-pass metabolism associated with oral delivery because the injected nanoparticles would pass through skin capillaries followed by systemic circulation, omitting portal circulation (Figure 1). Transdermal (TD) administration enables direct application onto or penetration into the dermal layers, facilitating effective localized tissue interaction or controlled release into the systemic circulation.

Although transdermal mRNA-LNP delivery and microneedle patches are being actively explored at the preclinical stage for immunotherapy, vaccines, and localized skin disorders, they have not yet become mainstream clinical products. LNPs enable the stable delivery of fragile mRNA through microneedle patches, and their skin permeability might be chemically optimized by modifying lipid composition [26]. However, challenges remain regarding potential skin irritation and the need to ensure long-term stability during mass production.

In contrast, EVs are extensively utilized in topical formulations such as creams, gels, and microneedles, focusing primarily on localized applications including skin rejuvenation, wound healing, and cosmetics. Preclinical studies have demonstrated the therapeutic potential of EVs for skin repair and regeneration, particularly through microneedle-assisted delivery [27]. Furthermore, they allow for non-invasive application via topical creams or patches.

**Figure 2:**
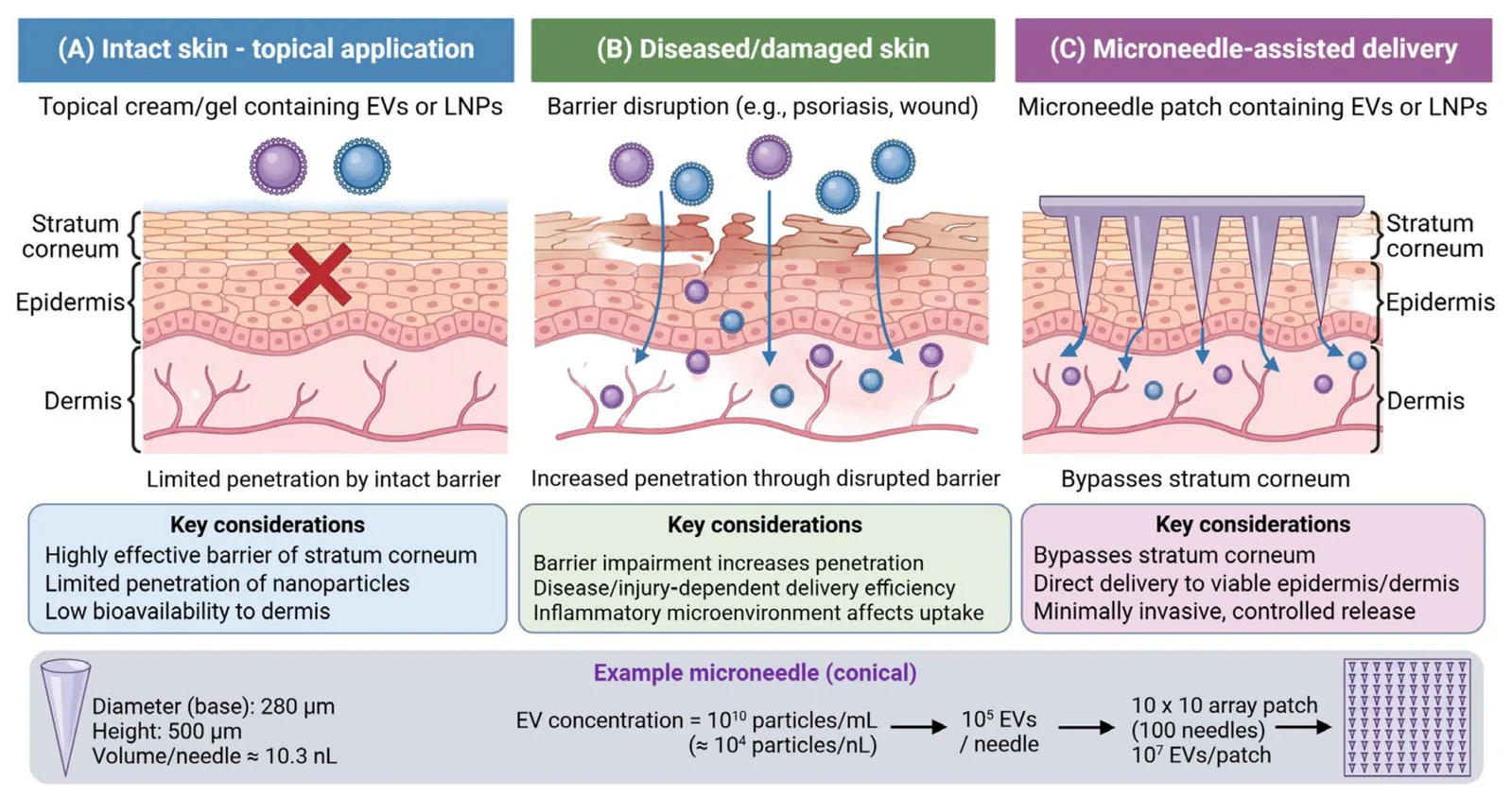
Biological and pharmaceutical considerations for transdermal delivery of EVs and LNPs. Comparison of EV and LNP delivery across intact, diseased/damaged, and microneedle-attached skin. (**A**) In intact skin, the stratum corneum acts as a highly effective physical barrier, substantially restricting the penetration of topically applied EVs and LNPs and consequently limiting their bioavailability in viable epidermal and dermal tissues. (**B**) In diseased or damaged skin, such as psoriatic or wounded skin, disruption of the epidermal barrier can facilitate nanoparticle penetration; however, delivery efficiency may vary depending on the extent of barrier impairment and the local inflammatory microenvironment. (**C**) Microneedle-assisted delivery physically bypasses the stratum corneum and enables direct deposition of EVs or LNPs into the viable epidermis and/or dermis. The lower panel illustrates a theoretical loading example for a conical microneedle (base diameter, 280 μm; height, 500 μm; calculated volume, approximately 10.3 nL). At an EV concentration of 10^10^ particles/mL, approximately 10^5^ EVs can theoretically be accommodated per needle, corresponding to approximately 10^7^ EVs in a 10 × 10 microneedle array. These values represent geometric estimates and do not account for formulation volume, encapsulation/loading efficiency, particle packing, or losses during fabrication and administration. Created in BioRender (https://www.biorender.com/).

The primary consideration in terms of cosmetics for patches containing exosome as a main ingredient is the skin penetration issue due to their size when applied to healthy skin. The diameter of exosomes ranges from 50–200 nm, which is unlikely to penetrate the tight junctions of the epidermis. Hence, microneedle-assisted pore formation on the epidermal layer is required in order to bring exosomes into the dermal layer. However, this approach can induce skin irritation and inflammation. Therefore, it would be more relevant to apply exosomes to conditions in which the barrier function of the skin is disrupted, such as Psoriasis or skin injuries (Figure 2A–C).

Second, from an industrial perspective, it would be important to calculate the theoretical number of LNPs or exosomes that one microneedle can contain. Assuming a hypothetical microneedle has a base diameter of 280 µm and a height of 500 µm, the volume of that microneedle is 10.3 nL.

(1) $$V=\frac{1}{3}\mathrm{\pi r}2h,\;r=140\;\mathrm{µm},\;h=500\;\mathrm{µm}$$

Assuming it can be completely filled with an exosome solution at a concentration of 10^10^/mL, this microneedle can hold 10^5^ number of exosome. If there is a 10 × 10 array microneedle patch, the total number is 10^7^. Of course, this calculation is highly hypothetical since, when preparing a polymer formulation, only a portion of the exosome stock would be added to the final mixture (Figure 2 bottom panel).

As a simplified theoretical exercise, the potential mRNA cargo capacity of an EV can be estimated using a volume-based approximation. The initial BNT162b2 mRNA from Pfizer/BioNTech was reported to consist of approximately 4000 nucleotides [28]. Since the average molecular weight of RNA is 330 Da/nt, we can take advantage of this to convert BNT162b2 mRNA to equivalent globular protein (1320 kDa). By further approximating the mRNA as an equivalent globular protein with a partial specific volume of 0.73 cm^3^/g, the volume of 4000 nt mRNA can be calculated as follows.

(2) $$V=\frac{\mathrm{MW}}{\mathrm{NA}}\times 0.73=\frac{1.32\times 10^{6}\;g/\mathrm{mol}}{6.022\times 10^{23}}\times 0.73\;{\mathrm{cm}}^{3}/g\approx 1600\;{\mathrm{nm}}^{3}$$

Importantly, this value represents a highly idealized, volume-based estimate rather than an experimentally achievable loading capacity. The calculation does not account for the complex conformation of mRNA, membrane thickness, endogenous EV contents, steric constraints, or the efficiency of cargo encapsulation.

Although this calculation does not reflect the actual mRNA loading capacity of EVs, it provides a theoretical benchmark for estimating cargo capacity and evaluating the feasibility of EV-based drug delivery. Furthermore, if the experimentally measured mRNA loading exceeds the theoretical estimate, potential measurement errors or artifacts should be carefully examined.

#### 2.1.6. Nasal Injection (IN)

Intranasal administration represents a highly promising, non-invasive strategy that enables direct drug delivery to the central nervous system (CNS) by bypassing the blood–brain barrier (BBB). This approach has gained significant traction due to its non-invasive nature and high patient compliance. Preclinical studies suggest that intranasal (IN) administration enables immediate deposition onto the nasal mucosa, facilitating efficient transport along the olfactory and trigeminal nerve pathways directly to the brain (Figure 1). However, the actual delivery efficiency of this route in humans remains largely unknown.

LNPs are being actively investigated via the IN route for targeting both CNS disorders and respiratory diseases, leveraging this BBB-bypass mechanism [29]. LNPs can be readily optimized for nasal spray formulations, allowing for precise dosage control. However, they carry risks of mucosal irritation and formulation leakage from the nasal cavity.

Similarly, an increasing number of studies utilize intranasal EV delivery to target brain diseases and modulate neuroinflammation.

#### 2.1.7. Ocular Injection

Ocular injection enables the localized delivery of therapeutics at high concentrations directly to target ocular tissues. Ocular delivery is utilized in targeting studies for retinal, macular, and corneal diseases, effectively bypassing the blood-retinal barrier (Figure 1). Intraocular administration includes intravitreal, subretinal, and suprachoroidal injection, enabling direct deposition into specific ocular compartments and potentially increasing local exposure to target tissues.

LNPs are primarily investigated for delivering gene and RNA therapies targeting retinal, macular, and corneal diseases, benefiting from this highly localized and concentrated delivery approach. LNPs offer precise gene delivery and demonstrate structural stability when injected intraocularly (e.g., into the vitreous humor). While LNPs provide robust nucleic acid protection within the vitreous humor, their clinical application requires careful optimization to avoid potential transient intraocular inflammation [30].

Concurrently, cell-derived EVs are actively being explored via both topical instillation and intraocular injection for applications such as corneal wound healing and retinal degeneration [31,32]. EVs provide inherent therapeutic benefits by promoting corneal and retinal regeneration, while exhibiting exceptionally low cytotoxicity.

**Figure 3:**
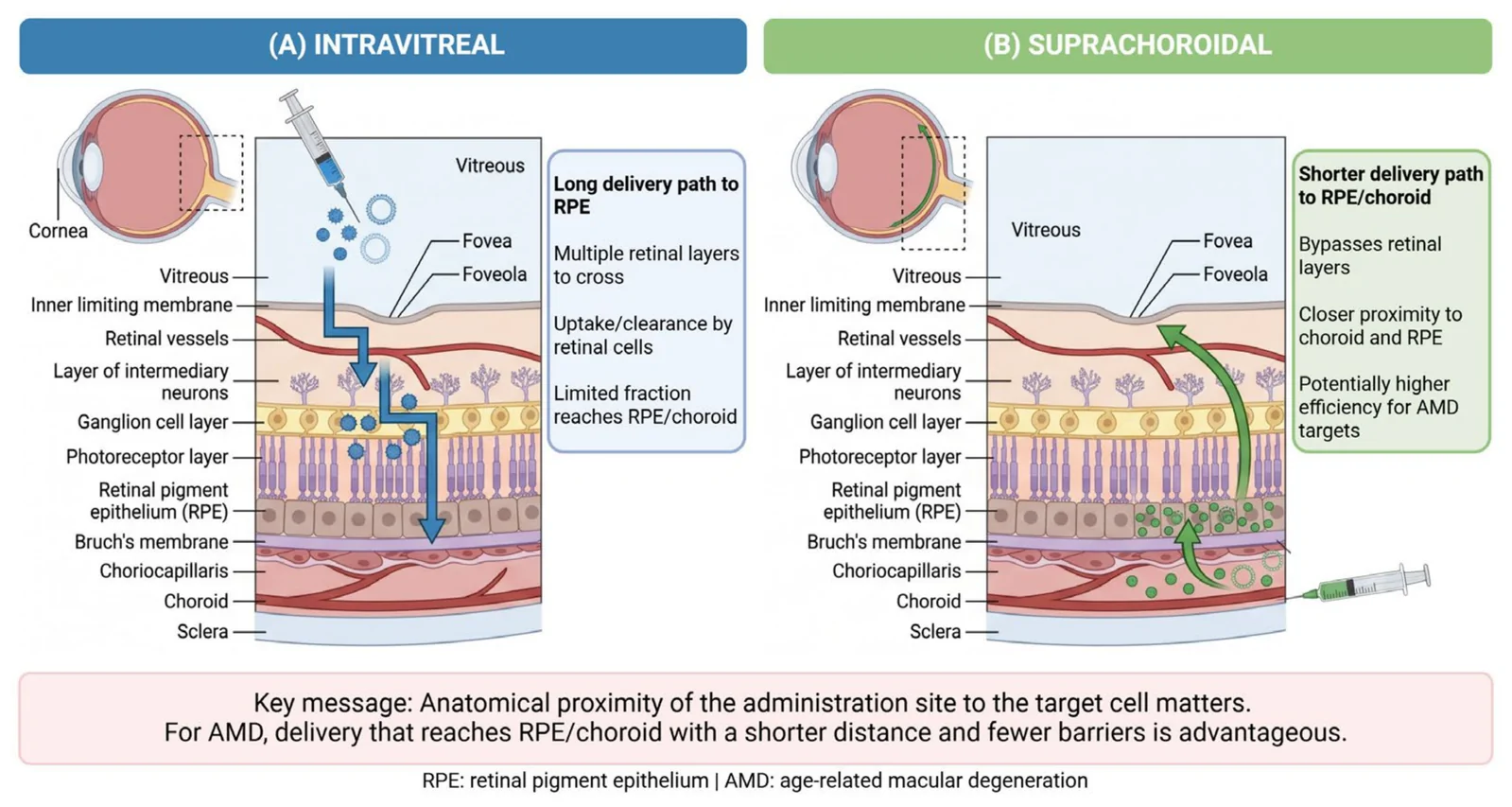
Anatomical considerations for EV and LNP delivery to the RPE in age-related macular degeneration (AMD). Schematic representation of the delivery pathways of EVs and LNPs following intravitreal and suprachoroidal administration to treat AMD. (**A**) Following intravitreal injection, EVs or LNPs injected would be first diluted with vitreous humor and must traverse the inner limiting membrane as well as multiple retinal layers before reaching the RPE and choriocapillaris. During this relatively long transport pathway, nanoparticle diffusion may be restricted by anatomical barriers, while cellular uptake and clearance within the retina may further reduce the fraction before reaching the RPEs. (**B**) Suprachoroidal administration deposits therapeutic nanoparticles in close proximity to the choroid and RPE, thereby providing a shorter delivery pathway and largely bypassing the intervening retinal layers. This anatomical proximity may improve local exposure of EVs or LNPs to the RPE/choriocapillaris and is therefore potentially advantageous for diseases primarily affecting the outer retina, such as age-related macular degeneration (AMD). RPE, retinal pigment epithelium; AMD, age-related macular degeneration. Created in BioRender (https://www.biorender.com/).

The intravitreal injection method is to inject the drug inside the vitreous humor (Figure 3A). Since the drug can be evenly dispersed inside the vitreous, the drug uptake is expected throughout the entire retina. However, it can increase ocular pressure when more than 1% volume of the vitreous is injected. It is widely agreed that human vitreous volume is about 5 mL. Therefore, one can calculate the number of LNPs or EVs per 50 μL to estimate the dose of API (active pharmaceutical ingredient). Moreover, intravitreal injection might not be the best approach to treat AMD (age-related macular degeneration) with LNPs or EVs since AMD arises in RPE (retinal pigment epithelium) or choriocapillaris, and LNPs or EVs need to travel through multiple retinal layers and microglia to reach RPE cells [33,34,35].

In this regard, suprachoroidal injection can be a better approach for AMD treatment (Figure 3B). Since the suprachoroidal space is in between sclera and choriocapillaris, LNPs or EVs travel a shorter distance to reach the target, potentially improving local drug exposure. Although currently approved anti-VEGF agents are injected into the vitreous, there have been reports of cases in which suprachoroidal delivery was considered [36]. In addition, unlike the intravitreal injection, the injection needle does not penetrate into the vitreous, ensuring higher patient compliance.

#### 2.1.8. Vaginal and Mucosal Delivery

Vaginal and other localized mucosal administration routes are increasingly recognized for their potential to induce mucosal immunity and treat regional disorders non-invasively.

Although research remains in the relatively early stages, LNPs are being actively investigated for delivering therapeutics and vaccines aimed at localized infections, hormonal therapies, and the induction of mucosal immunity. These studies primarily focus on conceptual frameworks for targeted drug and vaccine delivery within the mucosal microenvironment.

Similarly, EVs are utilized in exploratory and proof-of-concept studies targeting mucosal environments, with a particular focus on modulating neuro-inflammation, localized inflammatory responses, and the vaginal microbiota. Research is currently focused on leveraging the natural biocompatibility of EVs for microenvironmental regulation.

#### 2.1.9. Per Oral Lnjection (PO)

Oral administration represents a promising, non-invasive route due to its favorable patient compliance and ease of administration (Figure 1).

To overcome gastrointestinal barriers, extensive research is underway to develop LNP capsules or tablets engineered to withstand gastric acid and bile salts, with a focus on targeting intestinal epithelial cells and mesenteric lymph nodes [37]. While LNPs can be surface-engineered to target the intestinal epithelium specifically, protecting the encapsulated cargo from degradation by harsh gastric acidity and digestive enzymes remains a significant hurdle, limiting their current clinical translation.

Concurrently, oral delivery of dietary EVs—predominantly derived from milk or plants—is being actively investigated to modulate both intestinal and systemic diseases. These naturally occurring milk- and plant-derived EVs exhibit remarkable inherent resistance to gastric acid and demonstrate efficient, natural intestinal uptake. However, they often suffer from low systemic bioavailability following oral ingestion.

#### 2.1.10. Intrathecal Injection (IT)

Intrathecal (IT) administration offers a compelling strategy to circumvent the blood–brain barrier (BBB), enabling therapeutics to be delivered directly into the cerebrospinal fluid (CSF) for immediate access to the central nervous system (CNS) (Figure 1). This localized pathway allows the payloads to achieve therapeutic concentrations within the brain and spinal cord that are frequently difficult to attain via systemic routes, such as intravenous injection.

For lipid nanoparticles (LNPs), IT delivery serves as an efficient route to maximize local nucleic acid translation, as they are primarily internalized by neurons and astrocytes [38]. While LNPs require significantly lower doses through this route and minimize off-target organ toxicity, they may face limitations such as a potential risk of localized neuroinflammation triggered by synthetic components like ionizable lipids [39,40].

Similarly, EVs are actively investigated via the IT route due to their ability to diffuse through the CSF and leverage the glymphatic system to penetrate the brain parenchyma, effectively targeting neurons and glial cells. EVs provide excellent CNS bioavailability while minimizing systemic side effects; however, their therapeutic efficacy can be hindered by rapid clearance driven by physiological CSF turnover [41].

Furthermore, the highly invasive nature of this procedure for both delivery vehicles present inherent clinical risks, including infection and CSF leakage, and necessitates strict volume restrictions to prevent dangerous increases in intracranial pressure.

#### 2.1.11. Intratumoral Injection

Intratumoral administration directly targets the highly complex tumor microenvironment (TME), enabling most of the injected therapeutics to be delivered into the solid mass while bypassing systemic circulation. Hence, Intratumoral injection allows LNP or EVs to achieve high therapeutic concentrations within the primary tumor, effectively overcoming the physical barriers of dense desmoplastic tissue (Figure 1).

For lipid nanoparticles (LNPs), intratumoral delivery serves as a core platform for in situ vaccination, primarily utilizing mRNA-LNPs to express immunostimulatory cytokines (such as IL-12 or IL-27) locally to convert immunologically cold tumors into hot ones [42]. While intratumoral LNPs effectively induce robust local T-cell immunity and minimize severe systemic toxicities like cytokine storms, they are clinically restricted to palpable or image-guided accessible tumors, and synthetic lipids may occasionally induce local tissue necrosis.

Similarly, EVs are actively investigated via the intratumoral route to leverage their inherent biocompatibility and highly flexible lipid bilayers, which grant them superior penetration capabilities deep into the hard tumor stroma compared to rigid synthetic particles.

EVs provide excellent cellular uptake and can effectively deliver therapeutic cargos (e.g., oncogene-silencing siRNA) with low immunogenicity; however, their local efficacy can be hindered by rapid phagocytic clearance by tumor-associated macrophages (TAMs) [43].

Furthermore, the fundamental limitation of this highly localized procedure for both delivery vehicles is its inability to directly target undetected distant micrometastases, typically necessitating a combination with systemic therapies for comprehensive oncological management.

### 2.2. The Importance of Selecting Drug Administration Routes Based on Disease Type

The clinical success of an EV-based therapeutic is significantly influenced by its route of administration. This choice dictates the initial systemic concentration, the rate of clearance by the mononuclear phagocyte system (MPS), and the accumulation in target vs. off-target organs [10,19]. Therefore, the selection of a delivery route is not merely a logistical choice but a strategic decision based on the pathophysiology of the target disease and the presence of anatomical or biological barriers [1].

#### 2.2.1. Solid Tumors

Solid tumors present unique physiological hurdles, notably a dense tissue architecture, irregular vasculature, and high interstitial fluid pressure, which collectively restrict deep drug penetration into the tumor core.

Due to these disease-specific characteristics, relying solely on systemic intravenous (IV) administration often fails to deliver sufficient therapeutic concentrations to the center of the mass, despite its ability to target circulating tumor cells and micrometastases. Furthermore, aggressively increasing the IV dosage to overcome this pressure barrier is often associated with an increased risk of severe systemic toxicities, including immunosuppression and gastro-intestinal side effects.

Conversely, localized administration routes, such as intratumoral or intra-arterial in-jections, can maximize drug saturation within the primary mass to physically reduce the tumor burden while minimizing systemic exposure. However, local delivery remains ineffective against undetected distant metastases. Therefore, treating solid tumors effectively necessitates a strategic approach that combines systemic therapy to control micrometastasis with localized or actively targeted delivery systems to overcome the physical barriers of the tumor microenvironment (TME).

Exosomes demonstrate a distinct advantage in solid tumor therapy due to their inherent biocompatibility and flexible membranes, which grant them superior tissue penetration capabilities compared to synthetic nanoparticles.

This superior penetrability is actively being evaluated in clinical trials, such as the Phase 1 study of iExosomes at MD Anderson Cancer Center, which utilizes mesenchymal stem cell-derived exosomes to deliver siRNA into pancreatic cancer, one of the most highly desmoplastic and impenetrable solid tumors [44].

On the other hand, while lipid nanoparticles (LNPs) offer distinct advantages. in manufacturing scalability (CMC establishment) and encapsulation efficiency for genetic materials, their relatively rigid structure limits physical penetration into hard tumor tissues. Additionally, a significant portion of intravenously administered LNPs is prone to rapid clearance by macrophages or accumulation in the liver and spleen before reaching the tumor site.

To circumvent these physical and biodistributional limitations, current LNP-based solid tumor therapies often utilize systemic administration to stimulate a body-wide immune response rather than relying on direct tumor penetration, as demonstrated by BioNTech’s BNT111 (FixVac) RNA vaccine, which successfully drives potent T-cell immunity in melanoma patients [45].

#### 2.2.2. Alzheimer’s Disease and Neurodegenerative Disorders

In the treatment of neurodegenerative diseases such as Alzheimer’s disease (AD), the most formidable physiological obstacle is the blood–brain barrier (BBB), which strictly restricts the entry of therapeutics into the brain parenchyma. Due to this barrier, oral administration—while highly convenient for patients with cognitive decline—is largely ineffective for administering macromolecular biologics (e.g., antibodies, recombinant proteins, and RNA) due to the first-pass effect and their inability to cross the BBB.

Conversely, systemic intravenous (IV) injection can deliver large biologics into the bloodstream; however, typically only 0.1% to 1% of IV-administered antibodies success-fully penetrate the brain [46].

Consequently, achieving a therapeutically effective concentration in the CNS often requires relatively high systemic doses, which significantly increases the risk of severe adverse events, such as amyloid-related imaging abnormalities (ARIA), including brain edema and microhemorrhages.

To circumvent systemic circulation and directly access the brain, the intranasal (IN) route has been explored, utilizing the olfactory and trigeminal nerve pathways. While IN delivery offers a non-invasive method to bypass the BBB with minimal systemic side effects, its clinical application for high-dose drug delivery is heavily constrained by rapid mucociliary clearance and strict administration volume limits. Therefore, the current paradigm in AD treatment focuses on combining established administration routes with advanced nanocarrier-based drug delivery systems (DDS) to actively navigate or bypass the BBB.

EVs exhibit exceptional brain tissue penetrability and biocompatibility. For instance, in a preclinical study, modifying the EV membrane protein Lamp2b with an RVG peptide allows the vesicles to actively target and bind to neuronal receptors upon IV injection, facilitating BBB traversal [47]. The clinical viability of this systemic approach was demonstrated in a recent Phase I/II trial where intravenously administered allogenic human adipose mesenchymal stromal cell-derived exosomes (ahaMSCs-Exos) exhibited confirmed clinical safety in patients with mild to moderate AD [48]. However, the widespread clinical translation of EVs remains hindered by the extreme complexities and high costs associated with large-scale manufacturing and quality control (CMC).

In contrast, lipid nanoparticles (LNPs) offer unparalleled advantages: standardized mass production and high encapsulation efficiency for genetic materials (mRNA, siRNA). To cross the BBB, LNPs can be engineered for active targeting by conjugating ligands to their PEGylated surfaces, or they can rely on endogenous targeting, where blood-circulating ApoE proteins naturally adsorb to the LNP corona and subsequently bind to LRP1 receptors on brain endothelial cells. To overcome the inherent limitation of LNPs—namely, their propensity for off-target accumulation in the liver and spleen via macrophage clearance following IV injection—researchers are actively utilizing direct CNS administration routes. A prominent example is the ongoing Phase 1 study of Mivelsiran (ALN-APP), an RNA interference therapeutic for early-onset Alzheimer’s disease (EOAD), where intrathecal (IT) administration successfully bypassed systemic clearance, resulting in a dramatic 60–70% reduction in toxic amyloid precursor proteins within the cerebrospinal fluid [49].

#### 2.2.3. Psoriasis and Autoimmune Skin Disorders

Psoriasis is not merely a localized skin condition but a systemic autoimmune disorder characterized by the hyperactivation of immune cells, such as T cells, leading to the over-production of inflammatory cytokines and the abnormal, rapid proliferation of keratinocytes.

Consequently, the choice of administration route depends heavily on disease severity. For mild cases, topical administration (e.g., corticosteroid or vitamin D ointments) re-mains the first-line therapy, offering the advantage of minimal systemic absorption and low organ toxicity. However, its efficacy is limited in treating scalp or nail psoriasis, and the necessity of frequent application over large surface areas significantly reduces patient compliance. Furthermore, topical treatments are largely ineffective for moderate-to-severe cases covering more than 10% of the body surface area or when accompanied by psoriatic arthritis.

For such severe cases, oral administration is traditionally utilized to achieve systemic immune suppression. While convenient, conventional oral immunosuppressants broadly suppress the normal immune system and pose high risks of hepatotoxicity, nephrotoxicity, and gastrointestinal disturbances, generally precluding continuous long-term use (over one year).

To overcome these broad toxicities, the subcutaneous (SC) injection of targeted biologics has emerged as an attractive therapeutic route for moderate-to-severe psoriasis. SC administration allows for the precise neutralization of specific inflammatory cytokines without damaging normal immune cells or inducing liver and kidney toxicity. Further-more, the extended half-life of these biologics enables convenient self-injection via auto-injectors every 1 to 3 months. Nevertheless, this route is constrained by exorbitant costs and the risk of reactivating latent infections, such as tuberculosis or upper respiratory tract infections, necessitating rigorous pre-screening.

EVs are actively being explored as promising topical administration by leveraging their potent immunomodulatory and regenerative properties. Mesenchymal stem cell (MSC)-derived EVs, enriched with anti-inflammatory proteins and microRNAs, have been reported to suppress key inflammatory mediators like IL-17 and normalize keratinocyte proliferation in psoriasis models. Although EV penetration across intact skin remains limited, the compromised skin barrier in psoriatic lesions may facilitate enhanced epidermal and dermal delivery. The clinical potential of this non-invasive route was recently highlighted in a 2025 Phase 1 open-label study, which successfully confirmed the safety, tolerability, and excellent skin absorption of a topically applied MSC EVs ointment [50,51]. However, formulating these vesicles to maintain long-term stability without rupturing within an ointment base, coupled with high CMC costs, remains a significant pharmaceutical challenge.

Similarly, lipid nanoparticles (LNPs) are being investigated for the topical and transdermal delivery of nucleic acids (siRNA or mRNA) designed to silence specific psoriasis-inducing genes, such as STAT3 and TNF-α. Unlike conventional topicals that offer only temporary symptom relief, LNP-mediated intracellular delivery efficiently halts the production of pathogenic proteins by degrading the target mRNA. Moreover, LNPs offer the unique advantage of sophisticated lipid engineering; structural lipids with intrinsic anti-inflammatory properties can be incorporated to create “smart nanoparticles” that simultaneously provide gene therapy and inflammation suppression [52].

Despite these advanced capabilities, the synthetic ionizable lipids essential for LNP. formulation can inadvertently stimulate skin-resident immune cells, potentially causing pruritus or mild localized inflammation. Due to the need to perfectly resolve these poten-tial localized toxicities and lingering concerns over systemic side effects, LNP-based pso-riasis therapies have yet to reach large-scale Phase 3 clinical trials.

#### 2.2.4. Type I Diabetes Mellitus

Type 1 diabetes (T1D) is an autoimmune disorder characterized by the targeted de-struction of pancreatic beta cells, resulting in an absolute deficiency of insulin. Consequently, the primary therapeutic goal has traditionally been tight glycemic control via exogenous insulin administration. The standard of care relies heavily on multiple daily subcutaneous (SC) injections (MDI), which offer high accessibility and intuitive dose adjustments based on meal intake. However, MDI is fraught with limitations, including poor patient compliance, the constant risk of life-threatening hypoglycemia due to dosing errors, and the development of lipohypertrophy from repeated injections at the same site, which causes erratic insulin absorption.

To mitigate these risks, continuous subcutaneous insulin infusion (CSII) via insulin pumps, often integrated with continuous glucose monitoring (CGM) to form an automated insulin delivery (AID) system (or artificial pancreas), is strongly recommended. While AID systems drastically reduce nocturnal hypoglycemia, they carry a high economic burden and the critical risk of rapid-onset diabetic ketoacidosis (DKA) if the SC cannula occludes or the device fails.

Alternative routes, such as pulmonary delivery via inhaled insulin (e.g., Afrezza), provide a non-invasive option with a faster onset of action due to the vast alveolar capillary network. Nevertheless, precise micro-dosing is challenging compared to SC injections, and pulmonary delivery is contraindicated for patients with underlying respiratory conditions like asthma or COPD due to the potential for declining lung function and side effects like coughing.

In the pursuit of the advanced closed-loop system, microneedle array patches pre-loaded with glucose-responsive nanoparticles represent a paradigm shift. These “smart patches” chemically release insulin only when interstitial glucose levels rise, eliminating the risk of hypoglycemic shock. However, significant physical and physiological barriers hinder their clinical translation: the limited payload capacity of microneedles frequently fails to accommodate the large bolus doses required by T1D patients, and the inherent 10–15-min lag time in interstitial glucose levels compared to blood glucose delays rapid therapeutic responses [53,54,55]. Furthermore, the accumulation of synthetic polymer residues and glucose degradation byproducts within the dermal layers can trigger severe localized immune responses, including erythema and pruritus.

Beyond mere symptomatic glycemic control, next-generation nanomedicine aims to cure T1D by modifying the underlying autoimmune environment of the pancreas. Exo-somes, particularly those derived from mesenchymal stem cells (MSCs), are administered intravenously to achieve a dual therapeutic effect. Once delivered, anti-inflammatory miRNAs in EVs calm the autoreactive T cells attacking the pancreas, while growth factors in EVs promote the regeneration of surviving beta cells to restore endogenous insulin secretion. Following the demonstration of complete normoglycemia in animal models, early-phase clinical trials evaluating the safety of extracellular vesicle therapy for T1D are currently underway [56]. The primary hurdle for EV therapy remains the strict quality control required for CMC, as the quantity of active therapeutic components can fluctuate based on donor status and culture conditions.

Conversely, lipid nanoparticles (LNPs) are engineered for precision immune modulation, acting as “inverse vaccines” rather than traditional immune activators. By encapsulating specific autoantigen mRNAs within LNPs, researchers can induce antigen-specific immune tolerance, effectively instructing the immune system to cease its attack on pancreatic beta cells. A more recent LNP strategy involves directly protecting beta cells by transfecting them to express PD-L1—an immune evasion protein typically utilized by cancer cells—thereby creating a protective shield against T-cell-mediated destruction. As demonstrated in recent preclinical successes (e.g., Messenger RNA Delivery to Islet β cells Using Conjugated Lipid Nanoparticles), LNPs offer highly sophisticated engineering capabilities [57]. However, paradoxically, the synthetic ionizable lipids necessary for LNP formulation can inherently stimulate immune cells, potentially triggering unwanted systemic inflammation in a disease already characterized by immune hyperactivation.

### 2.3. The Therapeutic Efficacy of Drugs Based on Drug Administration Routes

The ultimate therapeutic efficacy and safety profile of a drug, particularly in the context of advanced nanocarriers like EVs and LNPs, are largely dictated by the chosen route of administration. The administration route determines not only the pharmacokinetic biodistribution pathway but also the specific types of immune cells the drug encounters first, thereby serving as a critical variable in modulating inflammatory responses and avoiding systemic toxicity.

Effective drug delivery requires matching the EV’s natural or engineered properties with the specific demands of the inflammatory microenvironment [58]. Current research focuses on balancing systemic reach with localized precision to minimize the “dilution effect” and off-target toxicity associated with conventional injectable therapies [59]. Since EV-based therapies have demonstrated remarkable outcomes in preclinical and early-phase clinical studies, where dysregulated immune responses drive pathology, in this review, we will focus more on immune-related diseases [58].

In this section, we will outline the factors to consider when deciding administration routes to achieve expected therapeutic efficiency.

#### 2.3.1. Anatomical Barriers and Transport Mechanisms

Specialized barriers—such as the BBB, the blood-cerebrospinal fluid barrier (BCSFB), the blood-retina barrier (BRB), and the intestinal mucosal barrier—regulate nutrient transport and homeostasis but also exclude over 98% of small molecule drugs and nearly all biologics [14]. EVs are uniquely capable of traversing these barriers via endocytic pathways, adsorptive transcytosis, or receptor-mediated transcytosis.

For CNS disorders, selecting a route that exploits these mechanisms is critical. While systemic administration may allow some EVs to cross a compromised BBB (as seen in acute stroke), chronic neuroinflammation often requires targeted engineering or the IN route to achieve therapeutic thresholds [8,60]. Furthermore, EVs can be transmigrated through endothelial cells into the brain parenchyma and then exit via the CSF, enabling a bidirectional flow that can be harnessed for both drug delivery and biomarker sampling [14,61]. Similarly, EVs are utilized in exploratory and proof-of-concept studies targeting mucosal environments, with a particular focus on modulating neuro-inflammation, localized inflammatory responses, and the vaginal microbiota. Research is currently focused on leveraging the natural biocompatibility of EVs for microenvironmental regulation.

#### 2.3.2. Influence of the Inflammatory Microenvironment

The efficacy of EV therapy is heavily influenced by the “licensing” effect of the local microenvironment. Mesenchymal stem cell-derived EVs (MSC-EVs) are known for their “smart” immunomodulatory properties, which are often activated or enhanced by the presence of pro-inflammatory cytokines such as TNF-α and IFN-γ (NCT06888973) [58].

In a non-inflammatory state, MSCs and their EVs might target neutrophils and monocytes with low efficacy. However, under acute inflammatory conditions, the polarized M1 macrophages “license” the EVs to inhibit effector T and B cells and natural killer (NK) cells, facilitating a shift toward a pro-healing M2 phenotype. Therefore, the timing and route of administration must align with the inflammatory peak of the disease to maximize this natural feedback loop.

The type of antigen-presenting cells (APCs) first encountered by the therapeutic vehicle can strongly influence the balance between immune activation and regulation. For instance, oral or mucosal administration introduces antigens to specialized dendritic cells within the gut-associated lymphoid tissue (GALT), a microenvironment that often exhibits a tolerogenic bias and can promote regulatory T cells (Tregs) response under non-inflammatory conditions.

Therefore, for selected mucosal immune disorders such as inflammatory bowel dis-ease (IBD), oral or localized mucosal administration may provide an advantage over systemic IV delivery by enhancing local tissue exposure and, in some contexts, promoting immune regulation while minimizing systemic inflammation.

#### 2.3.3. Organotropism and Cell-Source Tropism

The cellular source of the EV determines its intrinsic surface protein profile, which dictates its “organotropism” or natural homing tendency. For instance, melanoma cell-line-derived EVs (A375 EVs) show efficient tumor uptake, while macrophage-derived EVs exhibit high liver tropism [10]. This “like-targets-like” phenomenon can be exploited: cardiovascular progenitor cell-derived EVs are often used for heart failure therapies because they possess the necessary integrins to fuse preferentially with cardiomyocytes (NCT05774509).

#### 2.3.4. Impact of Biodistribution on Drug Clearance and Targeting

Fluorescent labeling is widely used to investigate EV biodistribution; however, lipophilic dyes such as PKH26 and DiR may dissociate from EV membranes, form aggregates, or persist after EV degradation, potentially leading to overestimation of intact EV accumulation in target tissues. Therefore, fluorescent signals should not be interpreted as direct evidence of intact EV distribution [62,63].

Fluorescent or bioluminescent reporter proteins, such as CD63-GFP and CD63-NanoLuc, enable tracking of EV-associated proteins with reduced dependence on exogenous membrane dyes. However, reporter stability, and signal persistence after EV degradation should be considered. Radiolabeling techniques, including positron emission tomography (PET) and single-photon emission computed tomography (SPECT), enable quantitative whole-body imaging and assessment of tissue distribution. Nevertheless, radiolabel dissociation or redistribution may complicate the interpretation of intact EV accumulation. Therefore, regardless of the tracking method, appropriate controls and validation of label stability, labeling efficiency, and EV integrity are necessary to ensure reliable biodistribution measurements.

Importantly, the detection of EVs in target organs does not necessarily indicate successful therapeutic cargo delivery. EV biodistribution, cellular uptake, and functional cargo delivery are distinct processes that should be evaluated separately. In particular, successful drug delivery requires intracellular cargo release and subsequent biological activity in target cells.

The divergence between vascular circulation and lymphatic absorption creates profound differences in drug efficacy. Intravenous (IV) administration introduces drugs directly into the systemic circulation, facilitating the rapid neutralization of inflammatory factors. This mechanism is highly advantageous for suppressing acute systemic inflammation, such as sepsis and cytokine storms, but is inherently limited by rapid phagocytic clearance driven by macrophages in the liver and spleen. Systematic reviews indicate that small EVs typically show peak detection in the liver and kidneys within the first hour post-administration, while localization in the lungs and spleen peaks between 2 and 12 h [4]. In contrast, large EVs demonstrate an immediate “first-pass” accumulation in the lungs before gradually redistributing to the liver over 24 h.

Conversely, subcutaneous (SC) injection of large biologics or nanoparticles primarily relies on slow absorption through the lymphatic system rather than blood capillaries. Since lymphatic vessels drain directly into lymph nodes densely populated with T and B cells, SC delivery is advantageous for directly targeting and suppressing active immune cells in chronic autoimmune diseases, including rheumatoid arthritis and psoriasis.

Intraperitoneal (IP) administration provides a distinct alternative, characterized by higher lymphatic uptake and more sustained release into the systemic circulation compared to IV. Small EVs delivered via the IP route show a temporal shift in organ distribution: at 24 h, the liver and gastrointestinal (GI) tract are the primary sites, but by 48 h, a significant accumulation occurs in the spleen and bone marrow. This suggests that the IP route may be particularly effective for therapies targeting the immune system or hematopoietic niches.

Intranasal delivery has emerged as a promising strategy for CNS disorders. Preclinical studies suggest that IN-administered EVs may reach the brain via olfactory and trigeminal pathways, potentially bypassing the BBB [64,65]. Some preclinical studies suggest that IN administration may increase cerebral exposure compared with IV administration while reducing peripheral distribution [66]. Recently, exosome delivery via the intranasal route has been extensively studied to treat ischemic injury, and 10^10^~10^11^ exosomes showed efficacy [67,68]. However, based on our search, there have been no reports that present quantitative analysis results showing the extent to which exosomes are delivered to the brain.

**Figure 4:**
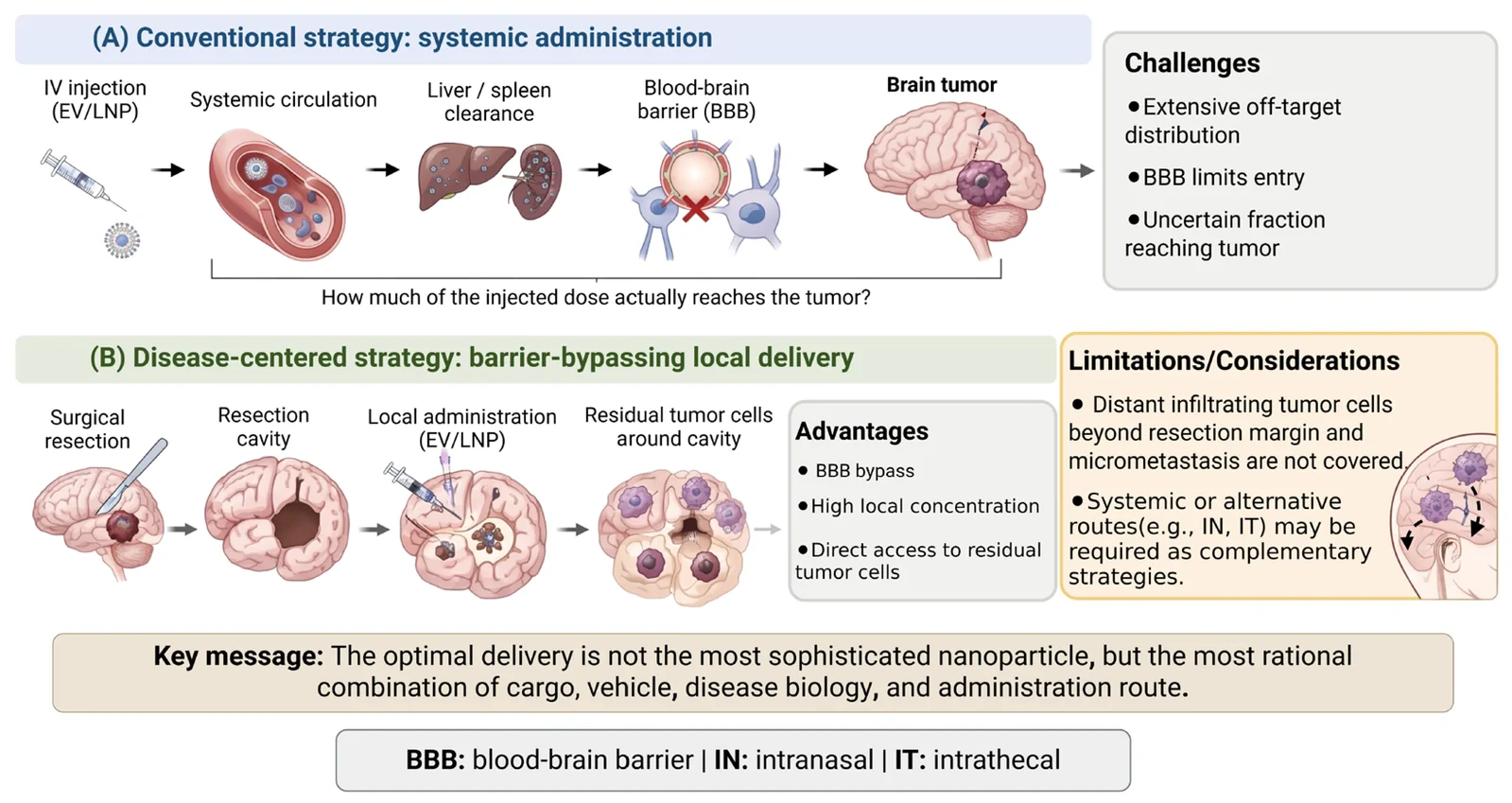
Disease-centered delivery strategies for EV-and LNP-based therapy of brain tumors. Strategy comparison of conventional systemic administration and local delivery strategies for EV- and LNP-based brain tumor therapy. (**A**) In case of intravenous administration, EVs and LNPs enter the systemic circulation, where substantial fractions may undergo off-target distribution and clearance by peripheral organs. The remaining particles must overcome the blood–brain barrier (BBB) before reaching the tumor, resulting in uncertainty of the dose that is ultimately delivered to the target site. (**B**) For brain tumors requiring surgical resection, the resulting resection cavity provides an opportunity for direct local administration of EVs or LNPs. This approach bypasses the BBB, enables high local exposure, and provides direct access to residual tumor cells surrounding the resection margin. However, local administration alone may not adequately target infiltrating tumor cells located beyond the resection margin or distant microscopic lesions. Systemic or alternative barrier-bypassing routes, including intranasal (IN) or intrathecal (IT) administration, may therefore serve as complementary strategies when broader CNS distribution is required. Overall, the optimal delivery strategy should be determined by integrating cargo properties, delivery vehicle characteristics, disease biology, and the anatomical accessibility of the therapeutic target rather than focusing exclusively on maximizing BBB penetration. BBB, blood–brain barrier; IN, intranasal; IT, intrathecal. Created in BioRender (https://www.biorender.com/).

For surgically resectable brain tumors, engineering EVs for BBB penetration or bypass may not always represent the most efficient delivery strategy. Surgical resection provides a unique therapeutic window in which EV- or LNP-based therapeutics can be administered directly into the resection cavity, thereby bypassing the BBB and achieving high local exposure to residual tumor cells [69]. Nevertheless, local administration cannot fully address diffusely infiltrating tumor cells located beyond the resection margin, highlighting a complementary role for systemic or intranasal delivery strategies capable of accessing anatomically distant tumor populations (Figure 4A,B).

The clinical feasibility of intranasal (IN) exosome administration has begun to be explored in humans. In a phase I/II clinical trial (NCT04388982), patients with mild-to-moderate Alzheimer’s disease received adipose-derived mesenchymal stromal cell exosomes at three dose levels (2 × 10^8^, 4 × 10^8^, or 8 × 10^8^ particles per dose) in a 1-mL formulation, administered intranasally twice weekly for 12 weeks. Repeated IN administration was generally well tolerated without treatment-related adverse events. Although modest improvements in cognitive measures, including ADAS-cog and MoCA-B scores, were observed, the small cohort size (*n* = 3 per dose group) and absence of a placebo control preclude meaningful conclusions regarding therapeutic efficacy. More importantly, from a drug-delivery perspective, the study provided no pharmacokinetic or biodistribution data. Thus, the absolute bioavailability and fraction of the administered dose reaching the brain remain unknown. Future clinical studies incorporating labeled-EV tracking, cerebrospinal fluid pharmacokinetics, or other quantitative biodistribution approaches will therefore be essential to determine the actual brain delivery efficiency and therapeutic potential of the IN route. For conditions such as brain tumors, where the brain is already accessible via surgery, a strategy of directly administering EVs or LNPs into the resection cavity to maximize local exposure may be far more logical than optimizing the intranasal (IN) route, for which even the brain delivery fraction is unknown. Conversely, for conditions such as Alzheimer’s disease (AD), which involve diffuse brain disease and where repeated intracranial administration is not practical, the value of the intranasal (IN) route becomes much greater.

Inhalation delivery such as nebulization or aerosolization is becoming the standard for respiratory inflammatory conditions such as ARDS or COVID-19-related pneumonia. This route ensures direct interaction with alveolar macrophages and epithelial cells, facilitating immediate anti-inflammatory action at the primary site of pathology [70]. The key factors will likely be the composition of the LNPs or EV-containing aerosol and the techniques to prevent the aggregation of EVs or LNPs during aerosol generation.

In the CNS, neuroinflammation is a “double-edged sword” that can promote repair in acute phases but leads to degeneration during chronic activation. Engineered EVs are being used to target the site of spinal cord injury (SCI) and reduce brain inflammation [60]. One innovative approach involves integrating the Angiopep-2 peptide into M2 microglia-derived EVs. These “M-Ang2-EVs” successfully target the blood-spinal barrier, promoting axonal remyelination and elongation while encouraging macrophages to phagocytose myelin debris. This level of precision—targeting specific cell subtypes like neurons and OPCs to transform them into repair-active phenotypes—is a hallmark of next-generation EV therapy.

Comparisons of EV delivery efficiency across administration routes are complicated by the lack of standardized dose reporting. EV doses are commonly reported as particle number, total protein amount, producing-cell equivalents, or therapeutic cargo amount, making direct comparisons between studies difficult. Furthermore, differences in administration volume and animal body weight can influence biodistribution and therapeutic outcomes. Therefore, consistent dose reporting and dose-normalized measurements, such as the percentage of administered dose per gram of tissue (%ID/g) and per organ (%ID/organ), are important for reliable comparisons across administration routes. These measurements should be accompanied by clearly defined sampling times and complementary dose information, including particle number, total protein amount, and therapeutic cargo content, where applicable. Importantly, these normalized values represent the distribution of the measured label or signal and should not automatically be interpreted as the fraction of intact EVs reaching the target tissue without appropriate validation.

#### 2.3.5. Direct Microenvironmental Control to Evade Systemic Toxicity

For highly localized inflammation confined by physical barriers, such as synovial fluid or cerebrospinal fluid, direct local administration via intra-articular or intrathecal injections is suggested. This targeted control allows for the delivery of high-concentration therapeutics directly into the pathological microenvironment.

Rheumatoid arthritis (RA) is characterized by chronic autoimmune-driven joint destruction. Clinical translation is currently focused on standardizing intra-articular injections to improve joint function and reduce radiographic damage [71,72]. EVs from various sources—most notably MSCs and M2 macrophages—exert therapeutic effects by reducing the secretion of pro-inflammatory cytokines (IL-6, TNF-α, IL-1β) and inhibiting the activation of the NLRP3 inflammasome [7,58,73]. Engineering MSCs to overexpress miR-320a produces EVs that specifically suppress the pathogenic functions of RA fibroblast-like synoviocytes (RA-FLS). Furthermore, BMSC-derived microvesicles deliver factors like PGE2 and TGF-β1 to promote the differentiation of regulatory B cells, establishing a tolerogenic immune environment.

In inflammatory bowel disease (IBD), intestinal homeostasis is disrupted by an abnormal immune response to gut flora. Current clinical investigations include the use of allogeneic bone marrow-derived MSCs (and their secretome) delivered via targeted endoscopic injection into the submucosal layer of the colon for medically refractory Crohn’s colitis (NCT04548583). This localized approach ensures a high concentration of therapeutic vesicles directly at the ulcerated tissue (NCT03609905). MSC-derived EVs offer a multi-pronged approach: they promote mucosal repair and modulate the intestinal immune cell dynamics toward a pro-healing phenotype [74,75,76]. Preclinical models of colitis have shown that EVs loaded with HIF1α or primed with IFN-γ significantly alleviate symptoms when administered via IP or local endoscopic injection.

Consequently, local delivery successfully circumvents systemic immune suppression and avoids the subsequent risk of fatal opportunistic infections, such as the reactivation of latent tuberculosis.

## 3. Discussion

The therapeutic potential of extracellular vesicles (EVs) and lipid nanoparticles (LNPs) has expanded rapidly, yet their clinical success will depend not only on the intrinsic properties of the delivery vehicle but also on whether the appropriate administration route is selected for the intended disease. As discussed throughout this review, each route creates a distinct pharmacokinetic and biological environment. Intravenous administration provides systemic accessibility but is accompanied by rapid clearance and substantial exposure to the liver and spleen; subcutaneous administration favors lymphatic transport; intranasal and intrathecal routes provide alternative access to the CNS; and intratumoral, intra-articular, ocular, and other localized routes can generate high drug concentrations within anatomically restricted pathological sites (Table 1). Therefore, there is unlikely to be a universally optimal administration route for EV- or LNP-based therapeutics. Rather, route selection should be regarded as an integral component of drug design and should be determined according to disease anatomy, target-cell localization, biological barriers, required dose, and the need for local versus systemic exposure.

An important future challenge is to move beyond qualitative descriptions of biodistribution toward quantitative assessment of delivery efficiency. The detection of EVs or LNPs in a target organ does not necessarily indicate therapeutically meaningful delivery. Parameters such as the fraction of the administered dose reaching the target tissue, cellular uptake by the intended cell population, retention time, cargo release, and functional intracellular delivery should therefore be quantitatively evaluated. This issue is particularly relevant for routes that are frequently described as highly efficient despite limited pharmacokinetic evidence. For example, intranasal administration is widely proposed as a non-invasive strategy for bypassing the BBB, but the actual fraction of administered EVs that reaches the human brain remains largely undefined. Similarly, for ocular, transdermal, and mucosal administration, anatomical proximity to the target does not necessarily guarantee efficient nanoparticle transport across intervening biological barriers. Future studies incorporating quantitative imaging, labeled-particle tracking, tissue pharmacokinetics, and cell-specific biodistribution analyses will be essential to establish realistic delivery efficiencies for each administration route.

Route selection should also reflect the specific pathological context rather than focusing exclusively on overcoming biological barriers. In surgically accessible diseases, for example, bypassing a barrier may be more practical than engineering nanoparticles to cross it. Following surgical resection of a brain tumor, direct administration of EVs or LNPs into the resection cavity may provide substantially higher local exposure to residual tumor cells than systemic or intranasal administration. In contrast, diffuse neurodegenerative disorders such as Alzheimer’s disease require repeated and widespread CNS exposure, making non-invasive approaches such as intranasal delivery or engineered systemic delivery more attractive. A similar principle applies to ocular diseases: for disorders involving the retinal pigment epithelium or choroid, selecting a route that deposits therapeutics closer to these tissues may be more rational than attempting to enhance penetration from the vitreous. Thus, future drug-development strategies should first ask where the therapeutic cargo needs to be delivered and which anatomical route provides the shortest and safest path to that target, before introducing additional nanoparticle engineering.

The optimal strategy may ultimately involve combining route selection with carrier engineering rather than treating these as independent variables. EV surface modification, LNP lipid composition, targeting ligands, controlled-release matrices, hydrogels, microneedles, and implantable or locally retained formulations could be tailored to the pharmacological requirements imposed by a specific administration route. For example, surface modifications, including the incorporation of targeting ligands, membrane fusion, and PEGylation, can influence EV circulation, biodistribution, and cellular uptake. For example, conjugation of rabies virus glycoprotein (RVG)-derived peptides has been investigated to enhance neuronal targeting, whereas iRGD peptides have been explored to facilitate tumor-associated targeting and tissue penetration [47,77]. Additionally, membrane fusion with synthetic liposomes can generate hybrid vesicles with tunable surface properties, while PEGylation may reduce nonspecific interactions and prolong systemic circulation. However, these modifications do not necessarily guarantee increased functional cargo delivery, and their benefits may depend on the administration route. Moreover, local and systemic administration should not necessarily be considered mutually exclusive. In solid tumors, for instance, local administration may maximize exposure within the primary lesion, whereas systemic delivery may simultaneously target circulating tumor cells and distant micrometastases. Such combination strategies could overcome the trade-off between local drug concentration and systemic coverage.

Clinical translation will additionally require route-specific consideration of manufacturability and dosing. For EVs, batch-to-batch heterogeneity, scalable production, cargo loading, storage stability, and potency assays remain major CMC challenges. For LNPs, inflammatory responses to ionizable lipids, off-target organ accumulation, and repeated-dose tolerability require continued optimization. Importantly, the feasible therapeutic dose is also constrained by the administration route itself. Limited volumes in the nasal cavity, vitreous humor, skin, or cerebrospinal fluid impose fundamentally different formulation requirements from those of systemic IV administration. Consequently, particle concentration, cargo molecules per particle, formulation volume, and the fraction of particles that actually reach the target should be considered together when determining whether a proposed delivery strategy is pharmacologically and industrially feasible.

An additional challenge in comparing EV delivery efficiency across administration routes is the substantial heterogeneity in experimental conditions and dosing regimens. EV doses are reported using different metrics, including particle number, total protein amount, producing-cell equivalents, and therapeutic cargo content, which are not directly interchangeable. Furthermore, variations in animal species, disease models, pathological severity, administration volume, dosing frequency, and sampling time can substantially influence the observed biodistribution and therapeutic outcomes. For example, a single intravenous administration and repeated intranasal administration cannot be directly compared without considering differences in cumulative dose, systemic exposure, and treatment duration. Therefore, comparisons across administration routes should ideally be performed using standardized EV preparations, harmonized dose metrics, and comparable experimental models. When direct head-to-head comparisons are unavailable, reported differences should be interpreted cautiously rather than attributed solely to the administration route.

In conclusion, the next stage of EV- and LNP-based drug delivery should shift from a carrier-centered paradigm toward a disease- and route-centered paradigm. Rather than asking simply how EVs or LNPs can be engineered to reach a particular organ, future development should determine which biological barriers truly need to be crossed, which can be bypassed through alternative administration routes, and how much of the administered therapeutic actually reaches the intended cells. Integrating disease pathology, anatomical accessibility, quantitative biodistribution, carrier engineering, and clinically feasible dosing will be critical for translating the promising biological properties of EVs and LNPs into reproducible therapeutic efficacy. Ultimately, the optimal drug delivery system will not be defined by the most sophisticated nanoparticle, but by the most rational combination of therapeutic cargo, delivery vehicle, disease biology, and administration route.

## Figures and Tables

**Table 1:** Summary of various administration routes for EVs and LNPs.

Route/ Target Tissues	EVs: Pros and Cons	LNPs: Pros and Cons	Major Barrier	Evidence (EV/LNP)
IV/Liver, tumors, systemic tissues	Systemic accessibility/Rapid phagocytic clearance, limited targeting	Established systemic RNA delivery/Liver accumulation, immune responses	MPS clearance, vascular barriers	Preclinical–early clinical/Clinical
IP/Peritoneum, abdominal tumors	Regional exposure/Lymphatic drainage, systemic absorption	Local RNA delivery/Clearance from peritoneal cavity	Peritoneal clearance, tumor penetration	Preclinical /Preclinical
SC/Lymph nodes, immune cells	Potential immunomodulation/Variable lymphatic uptake	Potential vaccine delivery/Local inflammation, formulation constraints	Extracellular matrix, lymphatic transport	Preclinical /Preclinical
IM/Muscle, APCs	Regenerative and vaccine potential/Limited clinical validation	Established mRNA vaccination/Local reactogenicity	Cellular uptake, local immune responses	Preclinical /Clinical
Transdermal/Skin, dermis	Potential tissue repair/Limited penetration through intact skin	Nucleic acid delivery via microneedles/Skin irritation, stability	Stratum corneum	Preclinical–early clinical/Preclinical
IN/CNS, nasal mucosa	Potential nose-to-brain delivery/Unknown human brain delivery fraction	Non-invasive CNS drug delivery potential/Mucociliary clearance	Nasal mucus, epithelial barriers	Preclinical–early clinical/Preclinical
Ocular/Retina, RPE, choroid	Potential regenerative effects/Limited penetration across retinal layers	Local RNA delivery/Inflammation, retinal barriers	ILM, retinal layers, ocular clearance	Preclinical /Preclinical
IT/CSF, CNS	Direct CSF exposure/Clearance by CSF turnover	CNS RNA delivery/Neuroinflammatory risk	CSF turnover, tissue penetration	Preclinical /Preclinical
Intratumoral/Solid tumors	Local cargo delivery/Limited distant tumor coverage	Local expression of therapeutic RNA/Restricted tumor accessibility	Dense tumor stroma, heterogeneous distribution	Preclinical–early clinical/Preclinical–clinical investigation
Oral/GI tract	Potential mucosal delivery/Variable stability and absorption	Engineered GI delivery/Gastric and enzymatic degradation	Gastric acid, mucus, intestinal epithelium	Preclinical /Preclinical

Abbreviations: IV, intravenous; IP, intraperitoneal; SC, subcutaneous; IM, intramuscular; IN, intranasal; IT, intrathecal; CNS, central nervous system; CSF, cerebrospinal fluid; RPE, retinal pigment epithelium; ILM, inner limiting membrane; MPS, mononuclear phagocyte system; APCs, antigen-presenting cells; GI, gastrointestinal.

## Data Availability

No new data were created or analyzed in this study. Data sharing is not applicable to this article.
